# *MPZL2*—a common autosomal recessive deafness gene related to moderate sensorineural hearing loss in the Chinese population

**DOI:** 10.1186/s12920-023-01786-3

**Published:** 2024-01-23

**Authors:** Lang Zhang, Jin-Yuan Yang, Qiu-Quan Wang, Xue Gao, Guo-Jian Wang, Ming-Yu Han, Dong-Yang Kang, Dong-Yi Han, Sha-Sha Huang, Yong-Yi Yuan

**Affiliations:** 1 College of Otolaryngology Head and Neck Surgery, National Clinical Research Center for Otolaryngologic Diseases, Sixth Medical Center of the PLA General Hospital, Chinese PLA Medical School, 6# Fucheng Road, Beijing, 100048 China; 2 State Key Lab of Hearing Science, Ministry of Education, Beijing, China; 3 Beijing Key Lab of Hearing Impairment Prevention and Treatment, Beijing, China; 4 Department of Otolaryngology, PLA Rocket Force Characteristic Medical Center, 16# XinWai Da Jie, 100088, Beijing, China

**Keywords:** Autosomal recessive inheritance, DFNB111, *MPZL2*, Moderate sensorineural hearing loss, Progressive hearing loss

## Abstract

**Background:**

Mutations in *MPZL2*, the characteristic genetic etiology of autosomal recessive deafness loci 111 (DFNB111), cause non-syndromic and moderate sensorineural hearing loss.

**Methods:**

In this study, we analyzed the phenotype and genotype of eight pedigrees consisting of 10 hearing loss patients with bi-allelic pathogenic or likely pathogenic variants in *MPZL2*. These patients were identified from a 3272 Chinese patient cohort who underwent genetic testing.

**Results:**

Apart from symmetrical and moderate sensorineural hearing loss, the *MPZL2*-related phenotype was characterized by progressive hearing loss with variation in the onset age (congenital defect to onset at the young adult stage). We determined that in the Chinese population, the genetic load of *MPZL2* defects was 0.24% (8/3272) in patients diagnosed with hearing loss and 7.02% (8/114) in patients diagnosed with hereditary moderate sensorineural hearing loss caused by *STRC*, *OTOA*, *OTOG*, *OTOGL*, *TECTA*, *MPZL2* and others. Three known *MPZL2* variants (c.220C > T (p.Gln74*), c.68delC (p.Pro23Leufs*2), c.463delG (p.Ala155Leufs*10)) and a novel start loss variant (c.3G > T (p.Met1?)) were identified. *MPZL2* c.220C > T was identified as the hotspot variant in the Chinese population and even in East Asia compared with c.72delA (p.Ile24Metfs*22) in European and West Asia through allele frequency.

**Conclusions:**

We concluded that apart from moderate HL, progressive HL is another character of *MPZL2-*related HL. No specified variant was verified for the progression of HL, the penetrance and expressivity cannot be determined yet. A novel *MPZL2* variant at the start codon was identified, enriching the variant spectrum of *MPZL2*. The hotspot variants of *MPZL2* vary in different ethnicities. This study provides valuable data for the diagnosis, prognosis evaluation and genetic counseling of patients with moderate sensorineural hearing loss related to *MPZL2*.

**Supplementary Information:**

The online version contains supplementary material available at 10.1186/s12920-023-01786-3.

## Introduction

An estimated 1.57 billion (95% uncertainty interval 1.51 ~ 1.64) people globally had hearing loss (HL) in 2019, accounting for one in five people. And By 2050, a projected 2.45 billion (2.35 ~ 2.56) people will have hearing loss, a 56.1% (47.3 ~ 65.2) increase from 2019 [1]. Genetic etiology can be found in about 60% of congenital hearing loss [2]. In terms of authoritative statistics, as of August 4, 2021 (last update), a total of 124 genes were reported to related to hereditary non-syndromic hearing loss on the website (https://hereditaryhearingloss.org/). However, according to the most recent literature report, more than 150 genes have been identified to link to hereditary non-syndromic hearing loss [3]. Only several genes have been clearly identified to be associated with mild-to-moderate HL [4]. It is crucial to determine the phenotype-genotype correlation of patients’ specific genetic mutations, which can not only predict and help in arresting the development of HL but also provide specific guidance for health management and family genetic counseling throughout the life cycle of patient [5]. In a word, an in-depth analysis of genetic and phenotypic data is necessary for determining the clinical characteristics of different types of genetic variations and help in patient-specific genetic counseling.

**Fig. 1:**
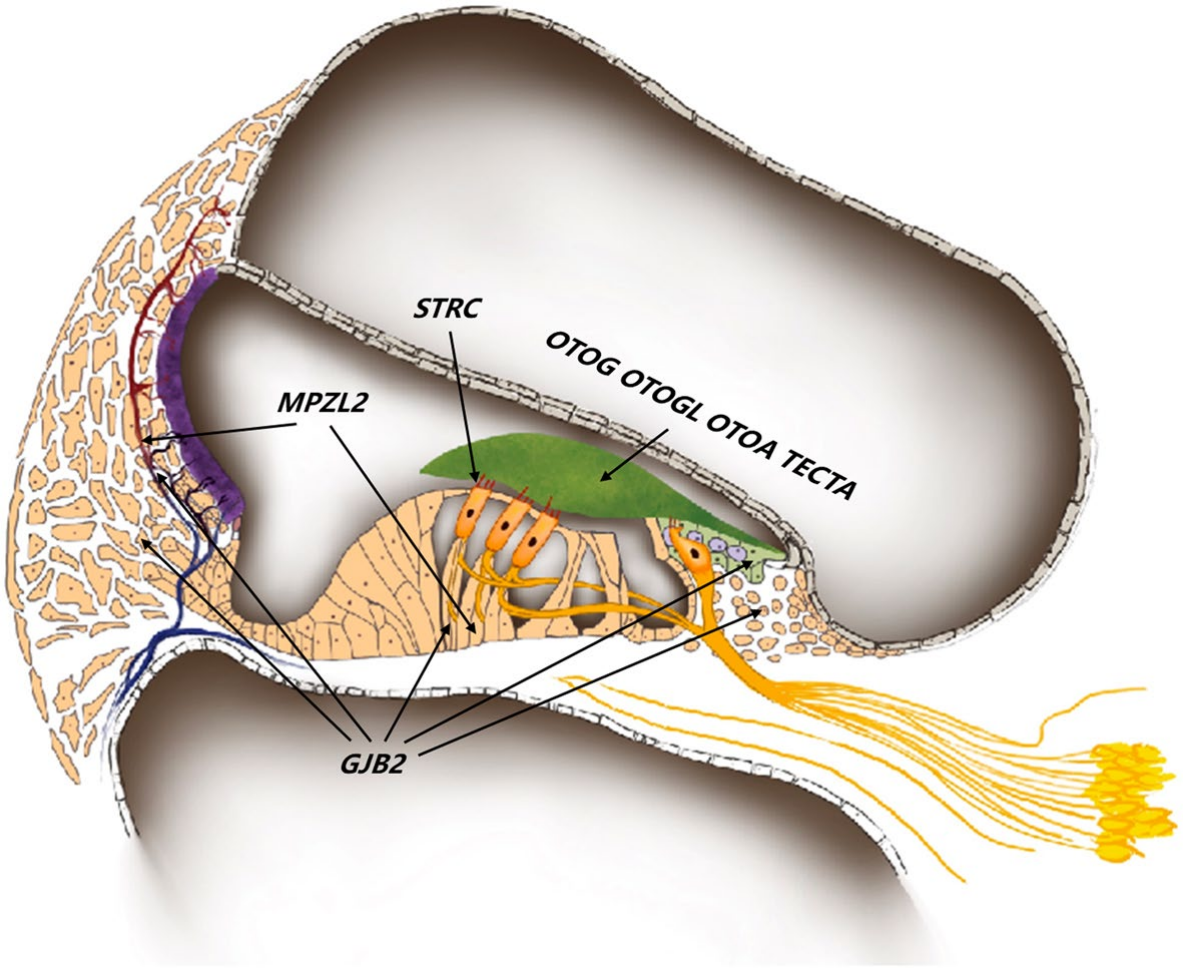
The location of *MPZL2*, *STRC*, *GJB2*, *OTOG*, *OTOGL*, *OTOA* and *TECTA* in the cochlea

Some genes closely related to mild-to-moderate HL have been reported, such as *Stereocilin* (*STRC*, MIM: 606440), *Otogelin* (*OTOG*, MIM: 604487), *Otogelin-like protein* (*OTOGL*, MIM: 614925), *Myelin protein zero-like 2* (*MPZL2*, MIM: 604873), *Otoancorin* (*OTOA*, MIM: 607038), and *Alpha-tectorin* (*TECTA*, MIM:602574) [6–11]. Common variant in *GJB2* can also cause mild-to-moderate HL, such as the p.V37I [12]. There exist similarities in the protein expression in the cochlea of *STRC*, *OTOG*, *OTOGL*, *OTOA* and *TECTA* [7, 11, 13]. Different from the most of the genes mentioned above, *MPZL2* expresses in the organs of Corti and the stria vascularis, especially in the basal regions of Deiter cells [8] (Fig. 1). However, there are relatively few reports on *MPZL2*.

Genetic defects in *MPZL2* were determined to be the cause of autosomal recessive deafness-111 (DFNB111, MIM: 618145) with non-syndromic, early-onset, symmetrical and moderate sensorineural HL, which was first identified by Wesdorp in 2018 [8]. The recent studies on DFNB111 have mainly focused on the Dutch, Moroccan, and some Middle Eastern countries [8, 14, 15]. In East Asia countries such as Korea [4, 16] and China [17], only a few families were identified with these defects and to date, no systematic phenotype-genotype correlation analysis for *MPZL2* has been performed.

In this study, by analyzing the genetic data of 3272 Chinese patients with HL, we determined the correlation between *MPZL2* defects and deafness, especially in those with mild-to-moderate HL. By analyzing the phenotypic and genotypic characteristic of these patients and reviewing the reported studies on *MPZL2* defects in other races, we determined the characteristics of deafness caused by *MPZL2* defects and attempted to provide precise guidance for the prognosis, treatment and family genetic counselling of the related hearing loss patients.

## Material and methods

### Ethical considerations

This study was approved by the Ethics Committee of Chinese PLA General Hospital (approval number S2018-088–01). Informed written consent was obtained from all subjects or their guardians for genetic analysis and the publication of the clinical data.

### Clinical data

Eight *MPZL2*-related HL pedigrees were enrolled in this study. The pedigrees were from a hearing loss patient cohort of 3272 different families, who underwent genetic testing from December 2015 to November 2022 in the Genetic Testing Center of Chinese PLA General Hospital. The medical history of each family member was obtained by using a questionnaire that included questions on the degree of HL, the age of onset of HL, the progression of HL, symmetry of HL, the use of hearing aids or cochlea implants, the presence of tinnitus and vertigo, infection, use of ototoxic drugs, noise exposure, and other relevant clinical manifestations to understand the otological manifestation and exclude any history of other diseases and environmental factors. Pure-tone audiometry with air and bone conduction was performed in accordance with the standard protocols in a sound-controlled room at frequencies ranging from of 125 ~ 8000 Hz. The patients underwent otoscopic examinations to evaluate the integrity of the tympanic membrane. High-resolute CT scans of the temporal bone were performed in some patients to exclude infection, space occupying lesions, and middle or inner ear malformations. If syndromic HL was suspicious, the probands underwent further general physical examinations, ultrasound of the heart, thyroid as well as other visceral organs, and craniocerebral MRI.

### Gene capture and next-generation sequencing (NGS)

Genomic DNA was extracted from the peripheral blood using a blood DNA extraction kit (TianGen, Beijing, China) in accordance with the manufacturer’s instructions.

Family 1 and family 3 were tested with deafness gene panels containing 415 known deafness genes, six mitochondrial regions associated with deafness, and three deafness-related microRNAs (Table S1), and other six families were tested with whole exome sequencing (WES). All coding exons, along with 100-bp flanking regions, were captured for each gene and then sequenced. The details of the deafness gene capture, sequencing, and bioinformatics analysis methods have been described previously [18]. Illumina NovaSeq6000 sequencing platform was used to conduct the WES. DNA samples from three *MPZL2*-related cases and their parents (family 2, family 6 and family 7) were subjected to trio WES, Sanger sequencing is used to verify the sequencing results and whether other family members carry the mutation. The nomenclature of the mutation described in Table 1 is based on *MPZL2* cDNA and protein accession numbers NM_005797.4 and NP_005788.1, respectively. We used the genomic coordinates from GRCH37/hg19 constructed from the human genome.

**Table 1:** Summary of *MPLZ2* variants and related phenotypes

**No**	**Family No. (patient No.)**	**Nationality**	**Allele 1**	**Amino acid change**	**Allele 2**	**Amino acid change**	**Age of onset (years old)**	**First time** **consultancy**		**Hearing** **follow-up**		**Phenotype**		**Pathogenicity**^**@**^ **(ACMG criteria)**
								**Age** **(years old)**	**PTA** **(dB HL)**	**Age** **(years old)**	**PTA** **(dB HL)**	**progression**	**symmetrical**	
1^Δ^		Dutch [8]	c.72delA	(p.Ile24Metfs*22)	c.72delA	(p.Ile24Metfs*22)	4	16	50.00	37	61.25	yes	yes	Pathogenic
2		Dutch [8]	c.72delA	(p.Ile24Metfs*22)	c.72delA	(p.Ile24Metfs*22)	9	22	47.50	42	68.75	yes	yes	
3		Dutch [8]	c.72delA	(p.Ile24Metfs*22)	c.72delA	(p.Ile24Metfs*22)	4	4	40.00	9	40.00	no	yes	
4^Δ^		Turkey [8]	c.72delA	(p.Ile24Metfs*22)	c.72delA	(p.Ile24Metfs*22)	3	5	37.50	8	42.50	no	yes	
5^Δ^		Turkey [8]	c.72delA	(p.Ile24Metfs*22)	c.220C > T	(p.Gln74*)	childhood	34	42.50	44	50.00	yes	yes	Pathogenic/ Pathogenic
6		Turkey [8]	c.72delA	(p.Ile24Metfs*22)	c.220C > T	(p.Gln74*)	3	6	37.50	13	36.25	no	yes	Pathogenic/ Pathogenic
7		Turkey [8]	c.72delA	(p.Ile24Metfs*22)	c.72delA	(p.Ile24Metfs*22)	5	5	26.25	16	42.50	yes	yes	Pathogenic
8		Turkey [8]	c.72delA	(p.Ile24Metfs*22)	c.220C > T	(p.Gln74*)	6	6	40.00	7	37.50	no	yes	Pathogenic/ Pathogenic
9^Δ^		Turkey [14]	c.72delA	(p.Ile24Metfs*22)	c.72delA	(p.Ile24Metfs*22)	Congenital or prelingual	**/**	**/**	21	R 46.25 L 48.75	no	yes	Pathogenic
10		Turkey [14]	c.72delA	(p.Ile24Metfs*22)	c.72delA	(p.Ile24Metfs*22)				14	R 55.00 L 50.00	no	yes	
11		Turkey [14]	c.72delA	(p.Ile24Metfs*22)	c.72delA	(p.Ile24Metfs*22)				8	R 48.75 L 51.25	no	yes	
12^Δ^		Turkey [14]	c.72delA	(p.Ile24Metfs*22)	c.72delA	(p.Ile24Metfs*22)				4	R 53.75 L 50.00	no	yes	
13^Δ^		Iran [14]	c.72delA	(p.Ile24Metfs*22)	c.72delA	(p.Ile24Metfs*22)	Congenital or prelingual	**/**	**/**	13	R 42.50 L 40.00	no	yes	Pathogenic
14		Iran [14]	c.72delA	(p.Ile24Metfs*22)	c.72delA	(p.Ile24Metfs*22)				8	R 35.00 L 35.00	no	yes	
15		Iran [14]	c.72delA	(p.Ile24Metfs*22)	c.72delA	(p.Ile24Metfs*22)				36	R 66.25 L 66.25	no	yes	
16		Iran [14]	c.72delA	(p.Ile24Metfs*22)	c.72delA	(p.Ile24Metfs*22)				5	R 70.00 L 97.50	no	no	
17^Δ^		Korean [4]	c.220C > T	(p.Gln74*)	c.463delG	(p.Ala155Leufs*10)	< 15	**/**	**/**	12.40 ± 4.20^#^	43.80 ± 8.10^#^	**/**	yes	Pathogenic/ Pathogenic
18^Δ^		Korean [4]	c.220C > T	(p.Gln74*)	c.220C > T	(p.Gln74*)						**/**	yes	Pathogenic
19^Δ^		Korean [4]	c.220C > T	(p.Gln74*)	c.220C > T	(p.Gln74*)						**/**	yes	
20^Δ^		Korean [4]	c.220C > T	(p.Gln74*)	c.220C > T	(p.Gln74*)						**/**	yes	
21^Δ^		Korean [4]	c.220C > T	(p.Gln74*)	c.220C > T	(p.Gln74*)						**/**	yes	
22^Δ^		Korean [4]	c.220C > T	(p.Gln74*)	c.220C > T	(p.Gln74*)						**/**	yes	
23^Δ^		Korean [4]	c.220C > T	(p.Gln74*)	c.220C > T	(p.Gln74*)						**/**	yes	
24^Δ^		Korean [4]	c.220C > T	(p.Gln74*)	c.220C > T	(p.Gln74*)						**/**	yes	
25^Δ^		Korean [4]	c.220C > T	(p.Gln74*)	c.220C > T	(p.Gln74*)						**/**	yes	
26^Δ^		Moroccan [15]	c.72delA	(p.Ile24Metfs*22)	c.72delA	(p.Ile24Metfs*22)	prelingual	/	/	childhood	R 49.17 L 33.75	**/**	yes	Pathogenic
27		Moroccan [15]	c.72delA	(p.Ile24Metfs*22)	c.72delA	(p.Ile24Metfs*22)					R 45.83 L 40.00	**/**	yes	
28^Δ^		Chinese [17]	c.220C > T	(p.Gln74*)	c.68delC	(p.Pro23Leufs*2)	13(R)	15	R 57.50 L 28.75	28	R 68.75 L 63.75	yes	no	Pathogenic/ Pathogenic
29^Δ^		Chinese [17]	c.220C > T	(p.Gln74*)	c.220C > T	(p.Gln74*)	congenital	0.25	R 25.00 L 35.00	1.25	R 40.00 L 45.00	yes	yes	Pathogenic
30^Δ^	Family 1 (II-1)	Chinese (this study)	c.220C > T	(p.Gln74*)	c.220C > T	(p.Gln74*)	15	15	R 42.25 L 43.25	32	R 60.00 L 61.25	yes	yes	Pathogenic
31	Family 1 (II-3)	Chinese (this study)	c.220C > T	(p.Gln74*)	c.220C > T	(p.Gln74*)	18	18	R 43.75 L 45.50	NA	NA NA	no	yes	Pathogenic
32^Δ^	Family 2 (II-1)	Chinese (this study)	c.220C > T	(p.Gln74*)	c.463delG	(p.Ala155Leufs*10)	19	19	R 47.75 L 50.00	20	R 48.75 L 50.00	no	yes	Pathogenic/ Pathogenic
33	Family 2 (II-2)	Chinese (this study)	c.220C > T	(p.Gln74*)	c.463delG	(p.Ala155Leufs*10)	12	12	R 43.75 L 48.75	NA	NA NA	no	yes	Pathogenic
34^Δ^	Family 3 (II-1)	Chinese (this study)	c.220C > T	(p.Gln74*)	c.3G > T	(p.Met1?)	childhood	NA	NA	38	R 57.50 L 56.25	yes	yes	Pathogenic/ Likely Pathogenic
35^Δ^	Family 4 (II-1)	Chinese (this study)	c.220C > T	(p.Gln74*)	c.68delC	(p.Pro23Leufs*2)	20	28	NA	35	R 65.00 L 63.75	yes	yes	Pathogenic/ Pathogenic
36^Δ^	Family 5 (II-1)	Chinese (this study)	c.220C > T	(p.Gln74*)	c.220C > T	(p.Gln74*)	21	21	NA	23	R 65.00 L 48.75	no	yes	Pathogenic
37^Δ^	Family 6 (II-1)	Chinese (this study)	c.220C > T	(p.Gln74*)	c.220C > T	(p.Gln74*)	7.5	13	R 43.75 L 38.75	16	R 61.25 L 61.25	yes	yes	Pathogenic
38^Δ^	Family 7 (II-1)	Chinese (this study)	c.220C > T	(p.Gln74*)	c.220C > T	(p.Gln74*)	10	10	R 42.50 L 43.75	14	R 42.50 L 45.00	no	yes	Pathogenic
39^Δ^	Family 8 (II-1)	Chinese (this study)	c.220C > T	(p.Gln74*)	c.220C > T	(p.Gln74*)	5	10	NA	10	R 42.50 L 41.25	no	yes	Pathogenic

*PTA* Pure Tone Average, / data not shown in literature, *NA* not available, *Δ* proband, *#* The data are shown as mean ± SD (Standard Deviation), *L* left ear, *R* Right ear, *@*—*MPZL2*:c.72delA (Pathogenic (PVS1, PP5_very strong, PM2)); *MPZL2*:c.220C > T (Pathogenic (PVS1, PP5_very strong, PM2)); *MPZL2*:c.463delG (Pathogenic (PVS1_strong, PP5_moderate, PM2_supporting, PM3)); *MPZL2*:c.68delC (Pathogenic (PVS1, PP5_moderate, PM2_supporting)); *MPZL2*:c.3C > T (Likely Pathogenic (PVS1_Moderate, PM2, PM3))

*Denotes gene truncation mutations, serving as a universal approach for denoting such mutations in written form, rather than relying on specific legends or notations

### Bioinformatic analyses

All detected variants were filtered against the 1000 Genomes Project, Exome Aggregation Consortium (ExAC), Human Gene Mutation Database (HGMD), and Exome Sequencing Project (ESP) databases. Changes with an unknown frequency or minor allele frequency < 1% were retained. Variants with no annotated frequency information in the databases were included. Variants predicted as low impact, missense changes predicted as not damaging by both SIFT and Polyphen-2, and synonymous variants were excluded. CNV Analysis along with both WES and targeted panel was conducted by use of Cnvkit software. The variants were comprehensively interpreted with the VarSome [19]. The manual variant classification was performed using the American College of Medical Genetics and Genomics (ACMG)/Association for Molecular Pathology (AMP) guidelines for genetic HL [20]. The final screened potential pathogenic variants were verified by Sanger sequencing and validated by parental testing on condition that the DNA samples of the parents were available. Novel variants were defined when it was absent form the HGMD professional database, ClinVar database, gnomAD database and published literature previouely.

The data, including phenotypes and observed variants, have been submitted to ClinVar (https://www.ncbi.nlm.nih.gov/clinvar/) under the accession numbers SCV002762660—SCV002762663.

### Sanger sequencing

Primers were designed for the sequence variations detected in *MPZL2*. Polymerase Chain Reaction (PCR) was used to amplify the corresponding DNA fragments of the probands and their parents, and Sanger sequencing was used to sequence the amplified products. The sequencing results were analyzed by DNASTAR (Madison) software. Primers sequences (reference genome: GRCh37/hg19) are as follows: *MPZL2* c.3G > T: upstream 5' AACCTGTTTGTCCGAGAGCAG 3'; downstream 5' AGTCACAGGCACAGGTGAGG 3'; *MPZL2* c.68delC: upstream 5' GCTCTGTCACTTGCTTTCCC 3', downstream 5' GAGGCGAGGCTTTAATGCTG 3'; *MPZL2* c.220C > T: upstream 5' TGCAGCTCTGTCACTTGCTT 3', downstream 5' GGGACAGATGCTCGGTTAAA 3'; *MPZL2* c.463delG: upstream 5' CTGCCCAGCCTTACGATTTT 3', downstream 5' AAGACACAGATTGCTCAGCC 3'.

## Results

### Clinical findings

**Fig. 2:**
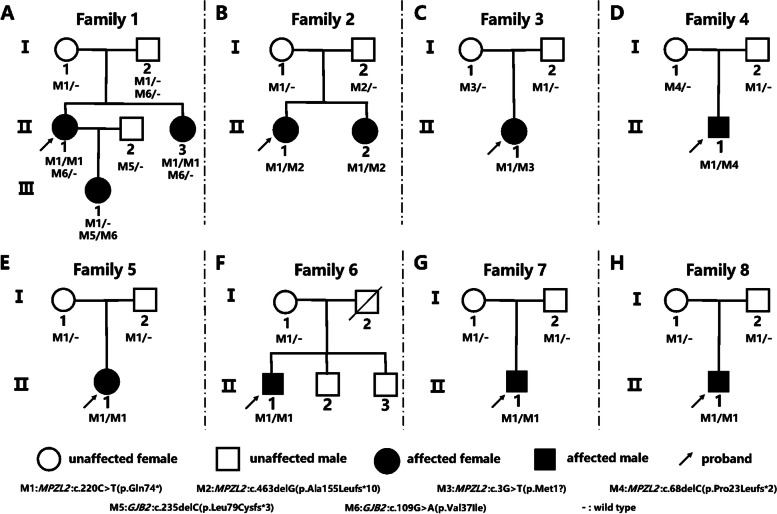
A Family tree of eight inherited HL pedigrees caused by bi-allelic variants of *MPZL2*. **A-H** The pedigree trees of Family 1 to Family 8, respectively. Six pathogenic or likely pathogenic variants including four in *MPZL2* and two in *GJB2* were identified in these families. All patients, except III-1 in Family 1, carried bi-allelic *MPZL2* variants. The variant of III-1 in Family 1 was identified as a carrier of *MPZL2*:c.220C > T(p.Gln74*) and compound heterozygotes of *GJB2* c.109G > A/c.235delC, suggesting that her HL is caused by *GJB2* mutations

Eight pedigrees with homozygous or compound heterozygous variants of *MPZL2* from a hearing loss patient cohort of 3272 who underwent genetic testing from December 2015 to November 2022 in the Genetic Testing Center of Chinese PLA General Hospital, were included in this study. There were totally 11 patients diagnosed as moderate sensorineural HL in the eight pedigrees (Fig. 2), among which the phenotype of 10 patients except for III-1 from Family 1 was confirmed to be *MPZL2-*related by next-generation and subsequent Sanger sequencing. III-1 in Family 1 carried heterozygous *MPZL2* c.220C > T (p.Gln74*) and compound heterozygous *GJB2* c.109G > A(p.Val37Ile)/c.235delC(p.Leu79Cysfs*3) inherited from her parents (Fig. 2). The HL level was classified according to 2021 WHO classification of HL (https://www.who.int/publications/i/item/world-report-on-hearing).

For the 10 patients carrying homozygous or compound heterozygous variants in *MPZL2*, progressive HL was reported by four patients (for more details please see Table 1). Moreover, we observed a 17.75 dBHL and 18 dBHL decrease in PTA of the right and left ears, respectively, during 17 years in patient no.30, and a 17.5 dBHL and 22.5 dBHL decrease in PTA of the right and left ears, respectively, during three years in patient no.37. The hearing level changes could not be determined because of the incomplete data for patients no.34 and 35.

The onset age of HL ranged from infant to 21 years, and the median age was 13.5 years. All but patient no.32 visited the hospital with HL as the first symptom. The main complaint of patient no.32 were the feeling of ear fullness and tinnitus but not HL. Interestingly, patient no.39 reported that he developed HL successively in either ear with a time interval of 5 years, and a subsequent hearing test conformed symmetrical moderate sensorineural HL in both ears. None of the patients complained of vertigo and hence the vestibular examinations were not performed.

### Detected variants of *MPZL2*

Four types of variants of *MPZL2* were detected in our study, including three truncation variants (*MPZL2*:c.220C > T (p.Gln74*), *MPZL2*:c.463delG (p.Ala155Leufs*10), and *MPZL2*:c.68delC (p.Pro23Leufs*2)), which result in the early termination of protein synthesis, and one start loss variant (*MPZL2*:c.3G > T (p.Met1?)). The start loss variant has not been reported previously and nor was it detected in the 686 individuals with normal hearing included in this study, but the three truncation variants are known pathogenic ones. The start loss variant was classified as likely pathogenic (PVS1_Moderate, PM2, PM3) according to the expert specification of the ACMG/AMP variant interpretation guidelines for genetic HL [20]. The allele frequency distribution of each mutation in different nationalities has been summarized in Table 2.

**Table 2:** The allele frequency distribution of *MPZL2* of each mutation in different nationalities

	Chinese	Dutch	Turkey	Iran	Korean	Moroccan
c.220C > T	24.36% (19/78)	-	3.84% (3/78)	-	21.79% (17/78)	-
c.72delA	-	7.69% (6/78)	19.23% (15/78)	10.26% (8/78)	-	5.13% (4/78)
c.68delC	2.56% (2/78)	-	-	-	-	-
c.463delG	2.56% (2/78)	-	-	-	1.28% (1/78)	-
c.3G > T	1.28% (1/78)	-	-	-	-	-

Allele frequency = number of alleles carrying each mutation/total number of alleles

The number of cases reported in this study and the literatures is 39, so the total number of alleles is 78 (39 × 2)

### Genetic load of *MPZL2* defects in the Chinese population with hearing loss

In a cohort of 3272 patients, 114 probands clinically diagnosed with moderate sensorineural HL were confirmed as hereditary by performing a genetic diagnosis. The related genes involved in the molecular pathogenesis of the 114 patients mainly included *STRC*, *OTOA*, *OTOG*, *OTOGL*, *TECTA*, *GJB2, KCNQ4, USH2A, PDZD7*, *MPZL2* and et al. *MPZL2* accounted for 7.02% (*n* = 8, 8/114) among the above genes resulting in a characteristic moderate HL. HL caused by *MPZL2* defects accounted for 0.24% (*n* = 8, 8/3272) of the Chinese population with HL.

## Discussion

At present, several studies on hereditary HL have been published, most of which have focused on congenital and severe-to-profound sensorineural HL. There are a few pathogenic genes associated with mild-to-moderate HL, and most patients with mild-to-moderate HL rarely opt for genetic counseling. Therefore, studies on the genetic factors causing mild-to-moderate HL are relatively less.

We retrospectively analyzed 3272 patients with HL who had undergone genetic testing from December 2015 to November 2022, and the results showed that *MPZL2* accounted for 0.24% of all 3272 patients diagnosed with HL. Furthermore, the molecular etiologies of 114 patients (3.48%) with mild-to-moderate HL were identified. The pathogenic genes consisted of *STRC*, *OTOA*, *TECTA, MPZL2, OTOG*, *OTOGL* and so on. Among them, *MPZL2* mutation was observed in 7.02% of patients with mild-to-moderate hereditary deafness, after *STRC*, *GJB2, OTOA* and *TECTA.* This finding is similar with the data (7.02%) reported by Kim [4], in whose study the related genes mainly included *STRC*, *OTOA*, *OTOG*, *OTOGL*, *TECTA*, *GJB2, KCNQ4, USH2A, PDZD7* and et al.. However, both our and the Korean studies [4] suggest the contribution of variations in *MPZL2* should not be ignored as the molecular genetic etiology in patients with mild-to-moderate sensorineural HL in East Asia.

In this study, 10 individuals from eight families were diagnosed with moderate HL caused by bi-allelic *MPZL2* variants. The following four pathogenic and likely pathogenic variations were involved: c.220C > T, c.463delG, c.68delC, and c.3G > T. The start loss variant c.3G > T, which may affect the initiation codon and translation of RNA, has been reported for the first time in this study. Another reported variant, c.72delA, was not found in our cases. So far, only five pathogenic variants were identified in *MPZL2*. Among them, 4 variants are truncation variants that could result in the early termination of protein synthesis, but it did not rule out the possibility that these variants would undergo nonsense-mediated decay (NMD). NMD is a quality control pathway that removes transcripts bearing premature termination codons (PTCs) unless PTCs are in the last exon and last ~ 50 nt of the penultimate exon [21]. Since *MPZL2* has six exons and the three truncation variants (c.68delC, c.72delA, and c.220C > T) are located in exons 2 or 4, these variants of *MPZL2* would likely undergo NMD, consistent with the evaluation of automatic PVS1 interpretation (AutoPVS1), an automatic classification tool (http://autopvs1.genetics.bgi.com/).

We also analyzed the allele frequency of all the reported variants. From the distribution of allele frequency, we concluded that c.220C > T and c.72delA are the most common variants of *MPZL2*. However, these two variants are distributed in different nationalities. *MPZL2* c.220C > T mainly occurs in East Asian populations, such as Chinese and South Korean, whereas c.72delA mainly exists in people with European ethnicities such as Dutch, Turkish and Italian. In the case of the Moroccan population, since only two cases were reported, its main variation cannot be accurately known yet. The other three variants (c.463delG, c.68delC and c.3G > T) were found only in Chinese and Korean patients. Since the number of reported cases is not large, we cannot regard them as unique variations only in the Chinese and Korean populations. For the common variations c.220C > T and c.72delA of *MPZL2*, previous studies also analyzed the founder effect. Haplotype analysis showed that the *MPZL2* c.72delA allele shared a common haplotype delimited by markers D11S1341 and D11S4104 in Dutch and Turkmen ethnicities from north-eastern Iran, and Turkish and Moroccan ethnicities [8, 14, 15]. Kim's study [4] showed that the *MPZL2* c.220C > T allele with an exclusively high minor allele frequency in the East Asian group of 1000 Genomes (0.0069) was carried by every patient of Han Chinese descent. Therefore, we believe that c.220C > T is a very ancient founding allele in East Asians and not just in Koreans. The results of the founder effect are consistent with our conclusion from the distribution of allele frequency.

At present, *MPZL2* gene is mainly associated with mild-to-moderate HL and progressive HL. These phenotypes are probably the result of the *Mplz2* variant that affects the adhesion of the inner ear epithelium, which leads to the loss of the structural integrity of the organ of Corti and the progressive degeneration of hair cells, supporting cells, and spiral ganglion neurons [8]. Moreover, the *Mpzl2* mutant mice showed early-onset progressive HL and a significantly increased ABR threshold [8], which was similar to the findings in human. However, the rate of HL development in the *Mpzl2* mutant mice was much faster than that in humans, and the HL in *Mpzl2* mutant mice rapidly developed to moderate and severe at 8 and 12 weeks, respectively (ABR threshold higher than 70 dB SPL) [8, 17]. To better demonstrated the extent of HL caused by *MPZL2* variants, we combined our results with those from previous studies (a total of 39 cases in Table 1) to comprehensively analyze the genotype and phenotype correlation of the *MPZL2* variant. The onset age of HL is wide, ranging from infant to 21 years, but is mainly early-onset. During the first consultation, all patients had moderate HL and could benefit from wearing hearing aids. In total, progressive HL was reported in four patients in this study and six patients in previous studies (Table 1). By comparing the hearing level at the first consultation and follow-up, we observed that 35.71% (10/28) patients had reduced hearing; however, the progression of HL could not be determined in 2 patients (patients no.34 and 35) in this study. The rate of HL was 2.10 and 13.33 dBHL per year in patient nos.30 and 37, respectively, which is higher than that reported in the literature (0.59 dBHL/year) [17]. A total of 64.29% (18/28) of patients did not report reduced hearing. Interestingly, all the audiograms of the subjects in our study showed higher thresholds in the high-frequencies (~ 4 kHz to 8 kHz) than in other frequencies (~ 0.125 kHz to 2 kHz), which could be partly explained by the histological abnormalities in the cochleae of the *Mpzl2*-mutant mice. The *Mpzl2*-mutant mice exhibited an altered organization of the outer hair cells and supporting cells, and degeneration of the organ of Corti, in addition to a mild degeneration of spiral ganglion neurons that was most pronounced at the cochlear base [8]. This conclusion needs to be confirmed by early diagnosis and long follow-ups.

In conclusion, in this study, a novel *MPZL2* variant at the start codon was identified, which enriched the variant spectrum of *MPZL2*. By analyzing the variant spectrum of *MPZL2* in the Chinese population, we further confirmed that c.220C > T has the founder effect on the East Asian population. The genotypic and phenotypic characteristics of *MPZL2* were analyzed more systematically by summarizing the findings of all the patients carrying *MPZL2* variants from this study and those reported in the previous studies, and we found apart from moderate HL, progressive HL is another character of *MPZL2-*related HL, which proposes the importance of pre-warning risk factors in order to arrest or put off the development of HL in patients. Thus, we believe that our findings have contributed to the early diagnosis of HL, prognosis evaluation, and could facilitate the genetic counselling of related patients.

### Supplementary Information

**Additional file 1: Table S1.** List of genes included in the deafness gene panels.

## Data Availability

The datasets presented in this study are available in the clinVAR (https://submit.ncbi.nlm.nih.gov/clinvar/).
