# Supplementary material for: MPZL2—a common autosomal recessive deafness gene related to moderate sensorineural hearing loss in the Chinese population
Source: BMC Med Genomics. 2024 Jan 23;17:32. doi: 10.1186/s12920-023-01786-3 (PMC10804618; doi:10.1186/s12920-023-01786-3)
Supplement: Supplementary file 1 — Additional file 1: Table S1. List of genes included in the deafness gene panels. [file 12920_2023_1786_MOESM1_ESM.pdf]

Table S1. List of genes included in the deafness gene panels

Deafness-related nuclear genes							
ABCD1	*ABHD12*	*ABHD5*	*ACO2*	*ACOX1*	*ACTB*	*ACTG1*	*ADCY1*
ADGRV1	*AIFM1*	*AK2*	*ALMS1*	*ALX3*	*ALX4*	*AMER1*	*ANKH*
ANKRD11	*AP1S1*	*ARSE*	*ASPA*	*ATP1A3*	*ATP2B2*	*ATP6V1B1*	*ATP6V1B2*
ATRX	*BCAP31*	*BCOR*	*BCS1L*	*BDP1*	*BEAN1*	*BMP1*	*BRAF*
BSND	*BTD*	*CABP2*	*CACNA1D*	*CATSPER2*	*CCDC50*	*CD151*	*CD164*
CDC14A	*CDH23*	*CEACAM16*	*CHD7*	*CHM*	*CHSY1*	*CIB2*	*CIDEA*
CISD2	*CLCN7*	*CLCNKA*	*CLCNKB*	*CLDN14*	*CLIC5*	*CLPP*	*CLRN1*
COCH	*COL11A1*	*COL11A2*	*COL1A1*	*COL1A2*	*COL2A1*	*COL4A3*	*COL4A4*
COL4A5	*COL4A6*	*COL9A1*	*COL9A2*	*COLEC10*	*COLEC11*	*COQ6*	*COX6B1*
CRTAP	*CRYM*	*DCDC2*	*DCHS1*	*DDX11*	*DHODH*	*DIABLO*	*DIAPH1*
DIAPH3	*DLX5*	*DMP1*	*DMXL2*	*DNA2*	*DNAJC17*	*DNAJC3*	*DNMT1*
DSPP	*DVL1*	*ECM1*	*EDN1*	*EDN3*	*EDNRA*	*EDNRB*	*EFTUD2*
EHMT1	*ELAC2*	*ELMOD3*	*ENPP1*	*EPS8*	*EPS8L2*	*ERAL1*	*ERCC6*
ERCC8	*ESPN*	*ESRP1*	*ESRRB*	*EYA1*	*EYA4*	*FAT4*	*FGF10*
FGF3	*FGF8*	*FGF9*	*FGFR1*	*FGFR2*	*FGFR3*	*FKBP10*	*FKBP14*
FLNA	*FLNB*	*FLVCR2*	*FOXC1*	*FOXI1*	*FREM1*	*FUCA1*	*FXN*
GAB1	*GALE*	*GATA3*	*GDF3*	*GDF5*	*GDF6*	*GFER*	*GIPC3*
GJA1	*GJB1*	*GJB2*	*GJB3*	*GJB6*	*GLYAT*	*GMPPA*	*GMPPB*
GNAI3	*GPC3*	*GPSM2*	*GRHL2*	*GRXCR1*	*GRXCR2*	*GSC*	*GSDME*
GSTP1	*GSTT1*	*GUCY2D*	*HARS*	*HARS2*	*HGF*	*HMX1*	*HOMER2*
HOXA1	*HOXA11*	*HOXA2*	*HOXB1*	*HPD*	*HSD17B10*	*HSD17B4*	*HSPA1A*
HSPA1L	*HSPA2*	*IARS2*	*IDS*	*IFITM5*	*IFNLR1*	*IGF1*	*IL13*
ILDR1	*IRX5*	*ITM2B*	*KARS*	*KAT6B*	*KCNE1*	*KCNJ10*	*KCNQ1*
KCNQ4	*KITLG*	*KRT9*	*LAMA3*	*LARS2*	*LHFPL5*	*LHX3*	*LMNA*
LMX1A	*LOXHD1*	*LRP2*	*LRP4*	*LRTOMT*	*MAF*	*MAN2B1*	*MANBA*
MARVELD2	*MASP1*	*MCM2*	*MEOX1*	*MET*	*MFN2*	*MGP*	*MIR182*
MIR183	*MIR96*	*MITF*	*MPZ*	*MPZL2*	*MSRB3*	*MYH14*	*MYH9*
MYO15A	*MYO1A*	*MYO1E*	*MYO3A*	*MYO6*	*MYO7A*	*NARS2*	*NDP*
NDRG1	*NEFL*	*NELL2*	*NF2*	*NLRP3*	*NOG*	*NOP56*	*NSD1*
OFD1	*OPA1*	*OSBPL2*	*OSTM1*	*OTOA*	*OTOF*	*OTOG*	*OTOGL*
P2RX2	*P3H1*	*PABPN1*	*PAX1*	*PAX2*	*PAX3*	*PCDH15*	*PCDH9*
PCNA	*PDE1C*	*PDSS1*	*PDSS2*	*PDZD7*	*PEX1*	*PEX10*	*PEX11B*
PEX12	*PEX13*	*PEX14*	*PEX16*	*PEX19*	*PEX2*	*PEX26*	*PEX3*
PEX5	*PEX6*	*PEX7*	*PHYH*	*PIGL*	*PJVK*	*PLCB4*	*PLEKHM1*
PLOD3	*PLP1*	*PMP22*	*PNPLA8*	*PNPT1*	*POLD1*	*POLG*	*POLG2*
POLR1C	*POLR1D*	*POU3F4*	*POU4F3*	*PPIB*	*PPIP5K2*	*PQBP1*	*PROK2*
PROKR2	*PRPS1*	*PRRX1*	*PTPN11*	*PTPRQ*	*PTPRR*	*RAB23*	*RAB40AL*
RAF1	*RDX*	*RECQL4*	*REST*	*RIPOR2*	*RNASEH1*	*RNASET2*	*ROR1*
RPGR	*RPS6KA3*	*RRM2B*	*S1PR2*	*SALL1*	*SALL4*	*SCO1*	*SEC23A*
SEMA3E	*SERAC1*	*SERPINB6*	*SERPINF1*	*SERPINH1*	*SETBP1*	*SIX1*	*SIX5*
SLC17A8	*SLC19A2*	*SLC22A4*	*SLC25A4*	*SLC26A4*	*SLC26A5*	*SLC29A3*	*SLC33A1*
SLC44A4	*SLC4A11*	*SLC52A2*	*SLC52A3*	*SLC9A1*	*SLITRK6*	*SMAD4*	*SMARCA4*
SMARCB1	*SMPX*	*SNAI2*	*SNX10*	*SOD1*	*SOD2*	*SOST*	*SOX10*
SOX2	*SOX9*	*SPARC*	*SPTBN4*	*SQSTM1*	*ST3GAL5*	*STRC*	*SUCLA2*
SUCLG1	*SYNE4*	*TBC1D24*	*TBX22*	*TCIRG1*	*TCOF1*	*TECTA*	*TFAP2A*
THOC1	*THRB*	*TIMM8A*	*TJP2*	*TMC1*	*TMEM126A*	*TMEM132E*	*TMIE*
TMPRSS3	*TMPRSS4*	*TNC*	*TNFRSF11A*	*TNFRSF11B*	*TNFSF11*	*TP63*	*TPRN*
TRIOBP	*TRMU*	*TSHZ1*	*TSPEAR*	*TUBB4A*	*TUBB4B*	*TWIST1*	*TWNK*
TXNL4A	*TYMP*	*TYR*	*UBR1*	*UGT1A1*	*UQCC2*	*USH1C*	*USH1G*
USH2A	*VHL*	*WBP2*	*WFS1*	*WHRN*	*XPNPEP3*	*YAP1*	

6 mitochondrial regions with deafness	Location
MT-RNR1	chrM:640-1601
MT-TL1	chrM:3230-3304
MT-CO1	chrM:5904-7445
MT-TS1	chrM:7446-7514
MT-TK	chrM:8295-8364
MT-RNR1	chrM:648-1601
MT-TA	chrM:5587-5655
MT-TC	chrM:5761-5826
MT-TQ	chrM:4329-4400
MT-TE	chrM:14674-14742
MT-TR	chrM:10405-10469

6 mitochondrial regions with deafness (Continued)	Location
MT-TF	chrM:577-647
MT-TS1	chrM:7446-7514
MT-TH	chrM:12138-12206
MT-TS2	chrM:12207-12265
MT-TI	chrM:4263-4331
MT-TT	chrM:15888-15953
MT-TK	chrM:8295-8364
MT-TW	chrM:5512-5579
MT-TL1	chrM:3230-3304
MT-TP	chrM:15956-16023

3 deafness-related microRNAs	Location
miR-96	chr7:129414532-129414609
miR-182	chr7:129410223-129410332
miR-183	chr:129414745-129414854
